# Nutraceuticals in the Mediterranean Diet: Potential Avenues for Breast Cancer Treatment

**DOI:** 10.3390/nu13082557

**Published:** 2021-07-26

**Authors:** Giuseppina Augimeri, Francesca Ida Montalto, Cinzia Giordano, Ines Barone, Marilena Lanzino, Stefania Catalano, Sebastiano Andò, Francesca De Amicis, Daniela Bonofiglio

**Affiliations:** 1 Department of Pharmacy, Health and Nutritional Sciences, University of Calabria, 87036 Arcavacata di Rende, Italy; giuseppina.augimeri@unical.it (G.A.); francescamontalto.93@libero.it (F.I.M.); cinzia.giordano@unical.it (C.G.); ines.barone@unical.it (I.B.); marilena.lanzino@unical.it (M.L.); stefania.catalano@unical.it (S.C.); sebastiano.ando@unical.it (S.A.); francesca.deamicis@unical.it (F.D.A.); 2 Centro Sanitario, University of Calabria, 87036 Arcavacata di Rende, Italy

**Keywords:** mediterranean diet, nutraceuticals, resveratrol, epigallocatechin gallate, retinoids, omega-3 polyunsaturated fatty acids, cell cycle, apoptosis, inflammation, angiogenesis

## Abstract

The traditional Mediterranean Diet constitutes a food model that refers to the dietary patterns of the population living in countries bordering the Mediterranean Sea in the early 1960s. A huge volume of literature data suggests that the Mediterranean-style diet provides several dietary compounds that have been reported to exert beneficial biological effects against a wide spectrum of chronic illnesses, such as cardiovascular and neurodegenerative diseases and cancer including breast carcinoma. Among bioactive nutrients identified as protective factors for breast cancer, natural polyphenols, retinoids, and polyunsaturated fatty acids (PUFAs) have been reported to possess antioxidant, anti-inflammatory, immunomodulatory and antitumoral properties. The multiple anticancer mechanisms involved include the modulation of molecular events and signaling pathways associated with cell survival, proliferation, differentiation, migration, angiogenesis, antioxidant enzymes and immune responses. This review summarizes the anticancer action of some polyphenols, like resveratrol and epigallocatechin 3-gallate, retinoids and omega-3 PUFAs by highlighting the important hallmarks of cancer in terms of (i) cell cycle growth arrest, (ii) apoptosis, (iii) inflammation and (iv) angiogenesis. The data collected from in vitro and in vivo studies strongly indicate that these natural compounds could be the prospective candidates for the future anticancer therapeutics in breast cancer disease.

## 1. Introduction

Nutraceuticals are food or parts of food which are considered complementary medicines in the field of food, food supplements, and pharmaceuticals [1]. In the last few years, nutraceuticals have received much attention because they have displayed health benefits and potential therapeutic actions in the treatment of several chronic diseases [2,3,4]. Among the most studied healthy dietary pattern, Mediterranean diet (MD) is a food model particularly enriched in nutraceutical foods referring to the eating behaviors of the population living in countries bordering the Mediterranean Sea in the early 1960s. The MD is characterized by prevalent consumption of fruits, vegetables, whole grain cereals, legumes, greater intake of olive oil, nuts, seeds, dairy products, low to moderate intake of fish, poultry, wine, little red meat consumption and low intake of sweets and processed foods. A key element of this food pattern is extra virgin olive oil, whose health benefits are derived from its unique composition, mainly enriched by high monounsaturated fatty acid (MUFA) content, notably oleic acid. In addition to MUFA, minor components of extra virgin olive oil, such as squalene, triterpenes, pigments, tocopherols or phenolic compounds entail antioxidant, anti-inflammatory, anti-tumor properties or act as a regulator of the intestinal microbiota [5,6]. Moreover, the high contents of bioactive compounds contained in many foods of MD, particularly polyphenols and vitamins in fresh vegetables and fruits or omega-3 polyunsaturated fatty acids (PUFAs) in fish and seeds, also protect cells by oxidative and inflammatory processes, inhibit carcinogenesis and induce antiproliferative effects in different types of neoplasia [7,8,9]. Indeed, approximately 30–35% of cancer cases are associated with dietary factors, highlighting clear evidence on the role of diet in controlling cancer, including breast carcinoma [10].

Breast cancer represents the most commonly occurring malignancy and the first leading cause of cancer-associated deaths in women worldwide. Based on WHO 2020 data, there were 2.3 million women diagnosed with breast cancer and 685,000 deaths globally [11]. The alarming incidence of breast cancer has led to a pressing demand in developing novel therapeutic strategies that could overcome the limitations of conventional therapies. Depending on the cancer stage, its treatment usually involves surgery, radiation and chemotherapy. Clinically, breast cancer is a heterogeneous disease that includes different molecular subtypes which can be identified through gene or biomarker expression analyses and are predictive of prognosis [12,13]. Breast cancer is classified as luminal-like breast cancer, which includes luminal A and B subtypes, characterized by the expression of estrogen receptor (ER) and/or progesterone (PR), and basal-like breast cancer, characterized by the absence of all three hormonal receptors (R-PR-Human Epidermal Growth Factor Receptor 2 (HER2)-triple negative breast cancer). Unlike ER-positive luminal tumors and HER2-positive tumors that show a more favorable prognosis partially due to anti-hormone therapy responsiveness, the lack of expression of the molecular targets is associated with the highest recurrence rate and the worst overall survival rate among all the breast cancer subtypes. Currently, strategies for breast cancer treatment depend on the evaluation of clinical and pathological parameters rather than gene expression array criteria. In particular, Ki-67 labeling index or histological grade have been proposed as important indicators in making the distinction between luminal A and B (HER2 negative) subtypes [14]. One of the most significant clinical problems in the treatment of breast cancer is that some patients relapse and/or eventually develop drug resistance. Thus, there is an urgent need to search for more effective and less toxic therapeutic strategies for breast cancer.

The present review focuses on current knowledge regarding the molecular mechanisms exerted by several nutraceuticals, such as resveratrol, epigallocatechin 3-gallate, retinoids and omega-3 PUFAs against typical hallmarks of breast cancer. Globally, research on compounds contained in MD is considered a promising field in breast cancer research to be further explored in clinical studies.

## 2. Search of the Literature

A PubMed search, run between 2000 and 2021, using the word “nutraceuticals and breast cancer” as the entry, yielded 1107 results, indicating that the scientific interest of nutraceutical area covered the oncology research field of the mammary gland over the last two decades. In the following text, some nutraceuticals included in the class of phytochemicals, vitamins or PUFAs—such as resveratrol, epigallocatechin 3-gallate, retinoids and omega-3 PUFAs that possibly affect breast tumor biology—will be reviewed and commented upon. Therefore, the data screened were organized in separate sections including (1) characteristics of nutraceuticals selected; (2) anticancer mechanisms of nutraceuticals on breast cancer related to (i) cycle growth arrest, (ii) apoptosis, (iii) inflammation and (iv) angiogenesis. Finally, due to the beneficial health effects of such molecules, we highlighted their potential as breast cancer preventive and/or therapeutic agents.

## 3. Nutraceuticals 

### 3.1. Resveratrol

**Figure 1:**
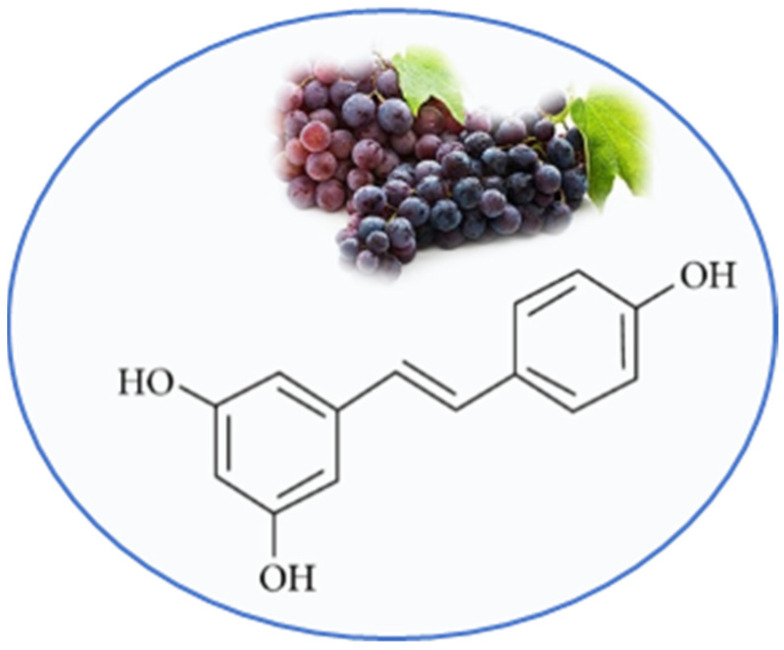
Chemical structure of resveratrol.

Resveratrol (RSV) or 3,5,40-trihydroxystilbene is a natural nonflavonoid polyphenol present in numerous plants. The primary sources of RSV are grapes skin and seeds, but commonly it is also detected in red wines [15]. RSV consists of two aromatic rings that are connected through a methylenic bridge (Figure 1). Two isomers are described: the trans-RSV form has the better stability and biological activity, the cis-RSV form is less active [16]. 

As a dietary compound, RSV is present as a glycosylated molecule, and glycosylation prevents the enzymatic oxidation of RSV, thus increasing its overall stability and bioavailability [17]. Glycosylated RSV analogs show similar biological activities upon transepithelial passage [18]. After oral ingestion, RSV amount, equal to 70–80%, is rapidly absorbed in the intestines [19] and after that, it is metabolized in the liver to glucuronide and sulfate forms. Trans-RSV peak is detected after 30–60 min post oral administration [20]. It is reported that in humans after a single 25 mg dose, there is a peak serum concentration of 2 mM (490 ng/mL) for RSV and all of its metabolites [19]. However, the remaining problems for using RSV in chemoprevention are the low bioavailability shown in animal and human studies and the consequent high doses of RSV required as reported in the majority of in vitro studies. RSV requires certain transporters for cellular extrusion. The vectoral transport of the glucuronidated and sulfated metabolites is mediated via transporters located in the basolateral and apical membranes of enterocytes, which notably are overexpressed in cancer cells. A huge amount of evidence has demonstrated that RSV plays a role in the prevention or delay of chronic degenerative diseases such as atherosclerosis, cardiovascular disease, type 2 diabetes and cancer [21,22]. In contrast, limited studies are dedicated to the evaluation of health benefits related to the consumption of phenolic compounds. The molecular mechanisms regulating RSV effects are extensively investigated in many studies, in which emerges a multitarget RSV action, affecting different signaling pathways [23]. Particularly, a large number of receptors, kinases and enzymes interact with RSV, mediating its biological effects. The principal mediators of RSV cellular effects are the sirtuin 1 (SIRT1) [24] and adenosine monophosphate-activated protein kinase (AMPK) [25], however, RSV has been shown to target steroid receptors [21]. It is a potent inhibitor of quinone reductase 2 activity, protein kinase C, aromatase, hetero-dimeric alphaVbeta3 integrin, multidrug resistance protein 1,13 β-lactoglobulin, human DNA topoisomerase II, which are some of the targets of RSV action. Furthermore, RSV triggers the expression of a wide range of antioxidant enzymes, determining an overall decrease in oxidative stress [26].

### 3.2. Epigallocatechin 3-Gallate

**Figure 2:**
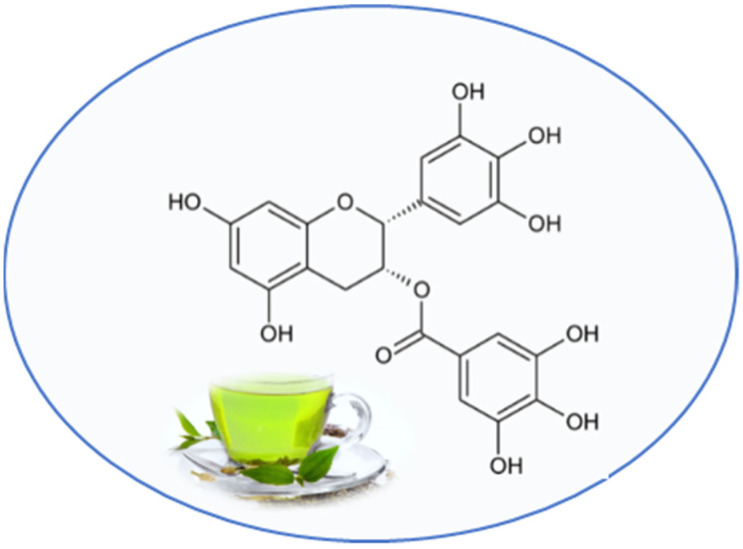
Chemical structure of epigallocatechin 3-gallate.

Epigallocatechin 3-gallate (EGCG) is contained in Green tea (Camellia sinensis), which comprises a large number of flavonoids, mostly catechins [27]. Compared to other tea catechins, galloyl moiety of catechins possesses the most biological activities (Figure 2). 

EGCG exhibits antioxidative, anticarcinogenic and antiproliferative action in several experimental models. In at least 13 animal models for human carcinogenesis of the lung, oral cavity, esophagus, stomach, small intestine, colorectal, colon, skin, liver, pancreas, bladder, prostate or mammary glands, EGCG has shown cancer preventive activities [28,29,30,31,32,33,34,35]. EGCG is first absorbed in the intestine where gut microbiota plays a critical role in the metabolism of EGCG. Several studies have reported that gut microbiota can deconjugate and degrade EGCG in vitro and in vivo. An interesting study indicates that in rats the radioactivity of isotope-labeled EGCG after oral administration starts to increase only upon 8 h [36], suggesting that EGCG undergoes extensive conversions by gut microbiota before absorption. At high doses and in certain environments, EGCG can act as a prooxidant owing to its auto-oxidation, resulting in the formation of the superoxide anion and hydrogen peroxide [37]. Such a prooxidant action of EGCG is viewed as a crucial anti-cancer mechanism. On the other hand, the antioxidative effects due to the specific polyphenolic structure are widely described [38,39]. EGCG is an antioxidant by direct trapping reactive oxygen species (ROS) or indirect induction of basal and/or nuclear factor E2-related factor 2 (Nrf2)-dependent antioxidant defense systems. In this concern, some data support the idea that a daily intake of green tea could reduce oxidative stress in vivo [40] suggesting that green tea principal components exert health beneficial effects, against cardiovascular disease, diabetes and cancer. Besides, EGCG has a positive effect on osteogenesis [41]. However, several studies regarding EGCG biological effects are still conflicting with quite a few controversies. Although there are multiple benefits associated with EGCG, several limits are still emerging. For example, EGCG shows low bioactivity via oral administration due to its oxidation, metabolism and efflux [42]. 

EGCG exerts its biological activities by binding to membrane components, including proteins and lipids. Moreover, EGCG regulates activities of cell surface growth factor receptors, especially receptor tyrosine kinases (RTK), including epidermal growth factor receptor (EGFR), vascular endothelial growth factor receptor (VEGFR), insulin-like growth factor receptor (IGFR) and the insulin receptor [43,44]. EGCG also increases other intracellular messengers including Ca^2+^, cAMP and cGMP. EGCG treatment increases cyclic adenosine monophosphate (cAMP) in endothelial cells and platelets, even though this effect is not observed in other cell types. The elevated cAMP stimulates protein kinase A causing various biological effects [45]. EGCG plays an important role in lipid metabolism by regulating lipolytic and lipogenic enzymes [46]. AMPK is an energy sensing molecule that is also activated by EGCG in hepatocytes, adipocytes, cancer cells and endothelial cells. AMPK contributes to inhibition of gluconeogenesis, stimulation of lipolysis, apoptosis, and reduction of endothelin-1 expression, respectively [47]. EGCG also modulates gene expression by inhibiting various transcription factors including Sp1, NF-κB, AP-1, STAT1, STAT3 and FOXO1 [48]. Multiple studies show that nuclear activities of EGCG inhibit inflammatory responses that are usually accompanied by increased oxidative stress [49]. 

### 3.3. Retinoids

**Figure 3:**
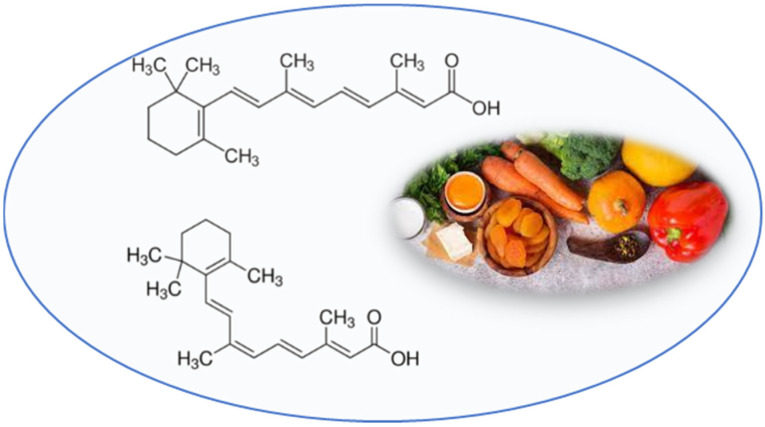
Chemical structures of all-trans-retinoic acid (**upper**) and 9-cis retinoic acid (**lower**).

Naturally occurring retinoids, all-trans-retinoic acid (ATRA) and 9-cis retinoic acid (9-cis RA), are structurally and functionally analogs of vitamin A (Figure 3).

They represent preformed vitamin A which is found in meat, poultry, fish and dairy products [50]. The recommended dietary allowance (RDA) for vitamin A is given as retinol activity equivalents (RAE) and a concentration of around 900 RAE in adults should be sufficient to meet the nutrient requirements of healthy individuals [51]. After ingestion and intestinal absorption by mucosal cells, retinoids are metabolized through several enzymatic processes and retinyl esters mainly stored in the liver. Retinol is released from retinyl esters in the blood and delivered to target tissues by retinol-binding protein (RBP). Both serum retinol and serum RBP are considered the gold standard for vitamin A status since these biomarkers are related to liver vitamin A concentrations [52]. Retinoids are involved in several important physiological processes such as cell differentiation and proliferation, embryonic development, reproduction, vision, maintenance of epithelial surfaces and immune competence. 

These compounds exert their biological activities by binding the two nuclear receptors called retinoic acid receptors (RARs) and the retinoid X receptors (RXRs). ATRA is the endogenous ligand for the RARs, whereas 9-cis RA binds both RARs and RXRs. Each receptor has three subtypes (α, β and γ), which are encoded by a single gene each. Both receptors are ligand-dependent transcription factors that as heterodimers bind to the retinoic acid receptor responsive element (RARE) and retinoid X receptor responsive element (RXRE), within the promoter regions of retinoid responsive target genes, regulating the gene expression [53]. However, RXRs can form heterodimers with other nuclear receptors, including peroxisome proliferator activated receptors (PPARs), also characterized by three subtypes (α, β and γ). Upon ligand binding, PPARs can modulate target gene expression by binding to the peroxisome proliferator responsive element (PPRE) in target genes [54]. Specifically, PPARγ activation inhibits tumor cell growth in vitro and in vivo model systems, suggesting the potential role of this receptor for cancer therapy [55,56,57,58]. Retinoids alone or in combination with PPARγ agonists are cell differentiation agents, which play a fundamental role in controlling cell proliferation and metabolism [59,60].

### 3.4. Omega-3 Polyunsaturated Fatty Acids

**Figure 4:**
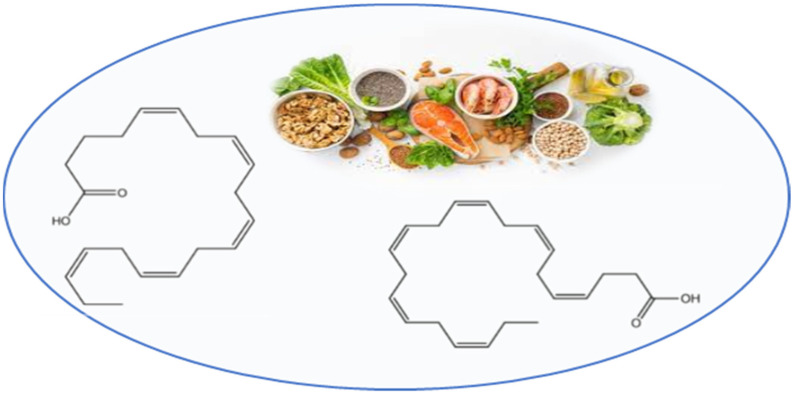
Chemical structures of eicosapentaenoic acid (**left**) and docosahexaenoic acid (**right**).

Omega-3 polyunsaturated fatty acids (PUFAs) including C20:5ω3 eicosapentaenoic acid (EPA) and C22:6ω3 docosahexaenoic acid (DHA) (Figure 4) naturally occur in highest quantities in cold water fish—such as salmon, tuna, sardines—and other seafood, like algae, as well as in fish oil supplements.

The global recommendation for EPA and DHA suggested the intake at least 500 mg/day of EPA+DHA in healthy adults [61,62] while omega-3 PUFAs should represent 1–2% of energy/day in general adult population [63]. PUFAs, which are incorporated into the membrane phospholipids, are responsible for numerous cellular functions including the maintenance of the cell membrane structure, fluidity, signaling and cell-to-cell interaction. These PUFAs have been shown to play important physiological roles in the cardiovascular, nervous, skeletal muscle systems, and in reducing inflammation. Blood levels of both EPA and DHA, which are dependent on their intakes, may be evaluated using the omega-3 index, reflecting the sum of omega-3 PUFAs in erythrocyte membranes as a percentage of total erythrocyte fatty acids. Low levels of omega-3 index have been associated with the highest risk for the development of inflammatory diseases [64]. The biological effects of DHA and EPA can also be exerted by modulation of cyclooxygenase (COX) activity, suppression of pro-inflammatory (NF-κ B), via modulation of toll-like receptor 4 (TLR4) signaling and activation of PPAR γ [65,66]. Furthermore, dietary intake of EPA and DHA have been shown to have cardioprotective, anti-inflammatory, immunoregulatory, antioxidant and anticancer activities [67,68,69]. Interestingly, growing evidence has highlighted that consumption of dietary omega-3 PUFAs stimulates the formation of omega-3 PUFA conjugates, such as the conjugates of EPA and DHA with ethanolamine, dopamine and serotonin that possess an increased biological activity compared to the parental compounds [70,71,72,73].

## 4. Anticancer Mechanisms

The hallmarks of cancer are acquired by defining capabilities that differentiate cancer cells from their normal counterparts and include sustaining cell proliferation and resistance to cell death. In addition, recent findings have highlighted the role of local chronic inflammation within the tumor microenvironment (TME) which leads to angiogenesis and increases the odds of metastasis.

### 4.1. Cell Cycle Arrest

Cell cycle dysregulation is a remarkable feature of tumor cells. For maintaining genomic integrity after damage to DNA, normal cells undergo cell-cycle arrest. At the molecular level, the cell cycle is governed by the temporally and spatially fluctuating activities of a cyclin-dependent kinase (CDK)/cyclin complexes. A complex network of events including binding of CDK inhibitors (CKIs) to the CDK/cyclin complex [74] are involved in the checkpoint mechanisms governing cell cycle phase transition. Aberrant cell cycle activity occurs either as a result of mutations in upstream signaling pathways or by genetic lesions within genes encoding cell cycle proteins. Dysregulation of CDKs, which contribute to tumorigenesis, provided a rationale for using compounds that inhibit CDKs as anticancer drugs.

#### 4.1.1. Resveratrol

It is well established that RSV can inhibit breast cancer cell growth in vitro and in vivo models by inhibiting cell cycle progression [75]. RSV can exert its effect at different stages of the cell-cycle [76]. Numerous studies investigated the cell-cycle proteins modulated by RSV and mediating its action. Several studies indicate the down-regulation of the cyclin D1/CDK4 complex by RSV in different cancer cell lines including breast [76,77]. However, RSV action on the cell-cycle is highly variable. RSV with other molecules classically used in breast cancer treatment show particularly marked effects inducing breast cancer cell cycle arrest [78], however the opposite action is reported depending on the RSV concentrations used. Precisely, RSV low dosage induces the proliferation of ERα+ breast cancer cells, although it is able to inhibit ERα- breast cancer cells [79]. Different RSV concentrations increased the population of G0/G1 phase with a concomitant decrease in the percentage of cells in S phase, suggesting a G1 arrest in tamoxifen resistant breast cancer cells. Authors indicated that the inhibition of cell cycle progression could be one of the events associated with the selective anti-proliferative efficacy of RSV in ERα+ and tamoxifen-resistant breast cancer cells. [80]. Similarly, Lee et al. set the concentration gradient in the range of 0–30 µM to study the effect of RSV on 4T1 breast cancer cells [81]. An elegant study investigated a total of 330 genes through large-scale transcriptome sequencing, including a number of upregulated genes and a major number of downregulated genes. Data obtained demonstrated that a relevant number of genes, differentially expressed after the treatment of RSV, were related to cell cycle regulation. Indeed, the cells were arrested in the S phase because the percentage of cells in S phase increased and cells in G1/G0 phase decreased. 

Further studies [82] using a genome-wide analysis of gene expression in MDA-MB-231 breast cancer cells indicate genes modulated by RSV. Interestingly, a significant decrease in the expression of genes involved in the cell cycle and DNA repair was evidenced. Moreover, the BRCA1 gene was upregulated, while two cell cycle regulators, cyclin D1 and cyclin B1 were repressed by RSV. Accordingly, authors indicated RSV impaired G1/S phase transition in both ERα- and ERα+ cells. These findings highlight the potential anticancer properties of RSV against breast cancer.

#### 4.1.2. Epigallocatechin 3-Gallate

Epigallocatechin 3-gallate was shown to be effective in the modulation of cell cycle regulators in different cell models including breast. EGCG was shown to be potent in breast cancer cell lines, but not in normal fibroblast WI-38. It specifically inhibited pro-survival genes [19]. Moreover, EGCG is currently under evaluation in several clinical trials for the treatment of several cancers [83,84]. 

In triple-negative MDA-MB-231 but also in ERα+ breast cancer cells, after prolonged exposure EGCG causes a significant reduction in DNA methyl transferase transcript levels [85]. Authors also reported that treatment resulted in complete demethylation of proapoptotic caspase recruitment domain protein, cyclin D2- and methyl-guanine methyltransferase (MGMT)-encoding genes in both cell lines. In the same models a higher dosage of EGCG for 9 or 12 days induces a significant inhibition of the telomerase reverse transcriptase hTERT, the catalytic subunit of the enzyme telomerase [86]. 

Interestingly, we discovered that EGCG inhibits breast cancer cell growth functioning as ERα down-regulator [87]. In these cells, ERα induces the cell cycle progression in a ligand-dependent fashion [88]. Our results suggest that potentiating EGCG/PR-B signaling should be further exploited for a clinical approach.

Treatment of non-obese diabetic mice with 0.2% EGCG added in their drinking water normalized the proportions of proliferating cell nuclear antigen (PCNA), a key factor in cell cycle regulation and Ki67-positive cells, and reduced the breast hypertrophy and hyperplasia [89]. Further studies [90] analyzed EGCG in combination with low dose nontoxic 5-aza-20-deoxycytidine (AZA) on the growth inhibition of breast cancer cells MCF-7, MDA-MB 231 and non-tumorigenic MCF-10A for 7 days. The results revealed the inhibition of breast cancer cell growth by co-treatment with AZA and EGCG compared to individual treatments, whereas no effect was observed in MCF-10A cells. The study indicates that potentiating the growth inhibition of breast cancer cells by AZA and EGCG combination treatment is, at least in part, mediated by epigenetic mechanisms. 

In ERα+ MCF-7 breast cancer cells the combination of EGCG, RSV and gamma-tocotrienol at suboptimal doses causes synergism in reducing cell proliferation, as compared to each of the three phytochemicals added alone. A significant effect was found at the expression levels of the bridging molecule cyclin D1 [91] and (B-cell lymphoma 2 (Bcl-2) expression after co-treatment. These results propose that diet-based protection against breast cancer may partially derive from synergy among dietary phytochemicals directed against specific molecular targets regulating cell cycle progression in breast cancer cells. These data support the development of a diet-based combinatorial approach in the prevention and treatment of breast cancer [92]. 

#### 4.1.3. Retinoids

Among several molecules able to impact the expression and activity of cell cycle regulators, ATRA is important in controlling the G1 transition to S phase in human breast cancer cells [93]. ATRA may also decrease the expression of cyclin D1 and D3 [94,95,96,97] and the activity of CDK2 and CDK4 [98]. CDK2 is a target for retinoic acid-mediated growth inhibition in MCF-7 human breast cancer cells [98]. ATRA induces hypo-phosphorylation of pRb and increases p21 levels [99] in breast cancer cells. Recently, it has been reported that ATRA treatment reduced both proliferation rates in T47D (ER+ and PR+) breast cancer cells markedly inducing cell cycle arrest [100]. Conversely, MDA-MB-231 breast cancer cells were unresponsive to ATRA exposure [100]. In three subtypes (ER+ MCF7, HER2+ SK-BR-3, triple negative HCC1806 and MDA-MB-231 cells) of human breast cancer cell lines, ATRA alone was not able to modulate cell cycle regulators. Instead, combination of omega-3 PUFAs/ATRA treatment significantly decreased the expression of p21 and p27 in both MCF7 and SK-BR-3 cells and markedly increased p21 and p27 expression in HCC1806 and MDA-MB-231 cells [101] addressing that combination of omega-3 PUFAs/ATRA affected cell cycle-related proteins in a cell specific manner. 

Furthermore, 9-cis RA decreased cyclin D1 and D3 expression levels as well as the expression and activity of CDK2 and CDK4 in breast cancer cells [96]. It also suppressed the growth of T47D breast cancer cells by G1 cell cycle arrest, through the Cyclin D1 and Cyclin D3 downregulation, which in turn causes Rb hypo-phosphorylation [102]. Either 9-cis RA or ATRA in combination with trastuzumab reduced cells in the S phase and enhanced G0/G1 phase of BT474 cells and the G2/M phase in SK-BR-3 cell [103]. Moreover, combined treatment with nanomolar levels of 9-cis RA and the PPARγ ligand, Rosiglitazone, induced an upregulation of p53 protein able to induce growth arrest in human breast cancer cells [104]. Thus, retinoids alone or in combined treatment with other agents appear to induce their anti-proliferative effects predominantly by blocking the transition from G1 to S cell cycle phase. 

#### 4.1.4. Omega-3 Polyunsaturated Fatty Acids

Most studies reported that the omega-3 PUFA DHA exerted G1 accumulating cells in breast cancer cells [105,106] although the mechanism(s) by which DHA exerts the effects are not fully elucidated. In MCF-7 breast cells, DHA induced G0/G1 cycle arrest by activating p53 and p21, while in SK-BR-3 cells inhibited proliferation by reducing ERK1/2 and STAT3 phosphorylation [107,108] and in MDA-MB-231 cells reduced the expression of Cyclin B1 [109,110]. Both EPA and DHA increased G2/M phase in MDA-MB-231 cells [110]. However, omega-3 PUFAs may also act in combination with anticancer agents to induce cell cycle arrest improving the efficacy of some drugs, such as doxorubicin [109]. In addition, the biological effects of DHA and EPA can also be exerted by their derivative molecules including DHA-dopamine (DHADA) and EPA–dopamine (EPADA) conjugates as well as DHA-ethanolamine (DHEA) and EPA-ethanolamine (EPEA) [111]. In particular, DHADA and EPADA arrested cell cycle and reduced concomitantly S-phase in MCF-7, SK-BR-3 and MDA-MB-231 cells, indicating that these molecules induced cell cycle arrest in breast cancer cells, independently of ER/PR/HER2 status [70].

### 4.2. Apoptosis Cell Death

Apoptosis or programmed cell death represents a key strategy for the elimination of neoplastic cells [112]. This tightly regulated cell suicide process is characterized by chromatin condensation, membrane blebbing, cell shrinkage, nuclear DNA fragmentation and the formation of apoptotic bodies which are rapidly recognized and engulfed by neighboring cells or macrophages [113]. Apoptosis can be triggered by the activation of the death receptors at the plasma membrane, as an extrinsic pathway, and through the activation of mitochondrial intrinsic pathway. Cell death signals stimulate tumor necrosis factor (TNF) family death receptors, such as TRAIL or CD95 (APO-1/Fas) receptors, which in turn activate the initiator caspase-8 and then executioner caspases-3, -6 and -7 that quickly begin to break down proteins leading to cell death. The stimuli that initiate the intrinsic pathway cause loss of the mitochondrial transmembrane potential and release of cytochrome c into the cytosol, where it binds to apoptotic protease activating factor 1 (APAF1) to form the apoptosome. At the apoptosome, the initiator caspase-9 is enabled leading to the activation of the executioner caspases-3 and -7 [114]. The regulation of these apoptotic mitochondrial events involves members of the Bcl-2 family of proteins that can be either pro-apoptotic or anti-apoptotic. Some of the anti-apoptotic proteins include Bcl-2, Bcl-x, Bcl-XL, and some of the pro-apoptotic proteins encompass Bax, Bak, Bid, Bad, Bim. These proteins can regulate the cytochrome *c* release from the mitochondria, triggering or blocking apoptotic process. Apoptosis evasion is a hallmark of all types of cancer and resistance to this cell death process and can augment the escape of tumor cells from surveillance by the immune system [115]. Targeting apoptosis is effective for breast cancer, thus many anticancer drugs that modulate both the intrinsic and extrinsic signaling pathways could potentially be used for cancer patients.

#### 4.2.1. Resveratrol

RSV exerts apoptosis in triple negative but also in ERα+ breast cancer cells [116] by different molecular links such as Src tyrosine kinase activity as well as the signal transducer and activator of transcription 3 (STAT-3) phosphorylation in breast cancer cells [117]. In addition, RSV produced pro-apoptotic signals by reducing p-AKT [118] but also by increasing phosphatase and tensin homolog on chromosome ten (PTEN) promoter activity [119].

Further studies demonstrated that RSV effectively enhanced the sensitivity of MCF-7 cells to adriamycin in a dose-dependent manner with the reduction of IC50 value and the increase of the number of apoptotic cells. The authors showed that RSV targeted miRNAs and their key target proteins. The RSV-induced chemosensitivity, and apoptosis were mediated by the modulation of the critical suppresser, miR-122-5p. Indeed, miR-122-5p inhibitors indicated a major effect of miR-122-5p on the regulation of key antiapoptotic proteins Bcl-2 [120,121] and cyclin-dependent kinases (CDK2, CDK4 and CDK6) in drug-resistant breast cancer cells in response to RSV. In addition, RSV induced apoptotic effects in MCF-7 and MDA-MB-231 cells by binding to the integrin αVβ3, activating ERK1/2 and p53-dependent pathway [122]. RSV action determines the nuclear accumulation of COX-2 which interacts with p53 and p300 promoting the apoptotic effect in MCF-7 and MDA-MB-231 cells [123]. In ERα+ cells, RSV promoted apoptosis in a caspase-independent pathway, whereas in triple-negative cells it enhanced the breakdown of mitochondrial membrane potential and the release of cytochrome *c* [124]. The apoptotic effect of RSV was observed to be blocked by Insulin-like Growth Factor 2 (IGF-II) in MCF-7 cells [125]. Trans-RSV mechanism can be determined by early production of reactive oxygen species (ROS) and lipid oxidation, later activation of c-Jun-N-terminal kinase (JNK) and p38 apoptotic signal [126]. A literature review revealed that several RSV analogs seem to be active in ERα- phenotypes, acting through an ER receptor-independent manner, inducing the expression of apoptotic genes. Interestingly, significantly lower tumor growth, decreased angiogenesis and increased apoptotic index in MDA-MB-231 tumors in RSV-treated nude mice was also shown compared with controls. Accordingly, in vitro studies showed a significant increase in apoptosis in RSV-treated MDA-MB-231 cells in addition to significantly reduced extracellular levels of VEGF [116].

#### 4.2.2. Epigallocatechin 3-Gallate

EGCG has remained an anticancer drug for its action mediated by the induction of the apoptosis. In ERα+ breast cancer (T47D) cells, EGCG decreased cell viability as concentration- and time-dependently (IC50 values 14.17 μM) [127]. Moreover, EGCG (80 μM) significantly increased the expression of proapoptotic genes such as PTEN [128], caspase 3, caspase 9 and decreased AKT approximately equal to tamoxifen. EGCG significantly increased Bax/Bcl-2 ratio and hTERT [127]. Authors based on bioinformatic tools, pathway enrichment and network analyses confirmed that Cyclin D1 overexpression, known for its oncogenic roles as regulator of cell cycle [91] and breast cancer aggressiveness [129], could be a target of EGCG. Particularly, bioinformatics analysis of microarray profiling identified the mechanism of focal adhesion kinase (FAK) signaling pathway in the apoptosis induction of breast cancer cells by EGCG [130]. EGCG inhibited miR-25 expression which plays a fundamental role in the development of breast cancer, it is also overexpressed in human breast cancer tissues and higher levels are detected in the serum of patients [131]. Besides, EGCG augmented Poly ADP-ribose polymerase (PARP), pro-caspase-3 and pro-caspase-9 at protein levels and restoration of miR-25 inhibited EGCG-induced breast cancer cell apoptosis. Similar results were reported in vivo models as evidenced by the reduction of Ki-67 and the increase of pro-apoptotic PARP expression. These results suggest that EGCG exerts chemopreventive action in breast cancer which may serve as a promising anticancer agent for clinical applications.

#### 4.2.3. Retinoids

One mechanism by which aretinoids exert their anticancer effects is through apoptosis induction [132]. ATRA may induce apoptosis in MCF-7 breast cancer cells after long-term exposure [133]. Even though ATRA triggered extrinsic apoptosis in multiple breast cancer cells through upregulation of death receptors and caspase activities, combined treatment of ATRA with TRAIL synergistically enhanced cell death [94]. Koay et al. reported that only the combination of the retinoids (ATRA or 9-cis-retinoic acid) with trastuzumab induced apoptosis in both ER+ and ER- human breast cancer cells with potential benefits for breast cancer patients [103]. We demonstrated that 9-cis-retinoic acid combined with Rosiglitazone (BRL) provoked intrinsic apoptosis in MCF7, SK-BR-3 and T47D breast cancer cells in a p53-dependent manner [104]. Moreover, independently of p53 transcriptional activity through the involvement of the p53/Bid/Bak multicomplex localized at the mitochondrial membrane of breast carcinoma cells [134]. On the other hand, omega-3 free fatty acids combined with ATRA synergistically triggered cell apoptosis via the activation of caspase signals without the involvement of p53 in multiple breast cancer cell lines [101]. Abdolahi et al. reported that DHA and ATRA provoked apoptosis in MCF-7 cells, indicating the usefulness of combined chemotherapy [135]. Several studies reported that DHA and EPA induced cancer cell death via apoptosis alone through different mechanisms [136,137,138,139] or in combination with conventional anticancer therapies [109,140]. For instance, omega-3 PUFAs chemically conjugated with other drugs, such as lovastatin, may provide novel therapeutic strategies for breast cancer [141].

#### 4.2.4. Omega-3 Polyunsaturated Fatty Acids

Multiple and complex modes of action are involved in the induction of apoptosis triggered by EPA and DHA in breast tumor cells in vitro and in vivo. Particularly, the proposed mechanisms of action of these compounds are: (i) incorporation into cell membranes, which causes the rearrangement of the signal molecules involved in the survival and death; (ii) production of elevated levels of intracellular oxidative stress; (iii) regulation of their metabolites; (4) binding to nuclear receptors, such as the tumor suppressor PPARγ. These main routes of action exerted by EPA and DHA may induce apoptosis in breast cancer cells and/or enhance sensibility of tumor cells to anticancer therapies [140]. Furthermore, omega-3 PUFA derivatives may exert their anticancer effects by binding to PPARγ [142]. It has been largely documented that the induction of extrinsic and intrinsic pathways of apoptotic process is a biological response resulting from PPARγ activation in breast cancer cells [143,144]. As conjugates of DHA and EPA, DHEA and EPEA elicit anticancer effects by PPARγ-mediated autophagy [71], while DHADA and EPADA triggered intrinsic apoptosis after long term treatment through PPARγ involvement in breast cancer cells [70].

### 4.3. Inflammation

Breast cancer cells engage in well-orchestrated reciprocal interactions with surrounding stromal and inflammatory cells to form an inflammatory TME that predisposes to the development of cancer and promotes all stages of tumorigenesis. Smoldering inflammation as a component of the TME is a recognized hallmark of cancer and if persistent promotes genetic instability [145]. Extensive investigations have uncovered the intrinsic pathway driven by genetic events causing neoplasia and the extrinsic pathway activated by inflammatory conditions inducing cancer [146,147,148]. Key molecules at the interplay between the intrinsic and extrinsic pathways include transcription factors and pro-inflammatory cytokines [149]. Unraveling the molecular pathways in the cancer-related inflammation could be essential for the further development of anti-cancer therapies.

#### 4.3.1. Resveratrol

RSV has demonstrated good potential as a cancer chemopreventive agent due to its established ability to attenuate the pro-inflammatory effects of obesity [150]. Specifically, RSV decreases TNF-α production [151], NF-κB activation [152] and COX-2 activity [153], critical mediators perpetuating the cancer-promoting effects of adipose dysfunction in obesity.

Zhu and his colleagues conducted an important study that assessed the effects of RSV on methylation of certain breast cancer-related proteins in women with increased risk [154]. A comparison was sought on the effect of either 5 or 50 mg of trans-RSV twice per day (for 12 weeks) on methylation of certain genes to those taking placebo. The results demonstrated increased levels of trans-RSV and RSV-glucuronide in circulation together with decreasing prostaglandin E2 (PGE2) expression in the breast [155]. It is important to underline that the disease progression from pre-cancer to cancer in the breast was associated with the increased levels of PGE2 together with the methylation of a protein similar to Ras effector protein (RASSF-1a) [156]. 

Adipose tissue dysregulation contributes to a chronic state of low-grade inflammation and is associated with increased risk and progression of several breast cancer subtypes, including claudin-low breast tumors. An elegant study demonstrated that RSV supplementation protected against serum metabolic and inflammatory perturbations in diet-induced obese (DIO) mice. Particularly, authors reported that RSV supplementation prevents obesity-induced adipose tissue dysfunction, mainly adipocyte hypertrophy, and reduces tumor growth in obese mice orthotopically transplanted with an aggressive claudin-low subtype of breast cancer. The mechanisms were related to: (1) smaller mammary adipocyte size; (2) decrease of expression of genes regulating inflammation promotion, macrophage recruitment and signaling; (3) reduction of the expression of COX-2 and pro-inflammatory eicosanoids in the mammary tissue; (4) changed systemic cytokines levels [particularly interleukin- (IL)-1β and IL-6]; and (5) maintenance of mammary expression of pro-adipogenic genes, notably PPARγ [157]. Further evidence, based on in-vitro and in-vivo studies, showed that RSV enhanced cancer chemotherapeutic potential of doxorubicin. This combination potentiates the effects of molecules on each other in synergistic manner and the acting on genes and proteins associated with inflammation (NF-κB, COX-2) and autophagy (LC3B, Beclin-1). Thus, these findings suggest not only the crosstalk of signaling pathways associated with chronic inflammation, autophagy and apoptosis altered during breast cancer pathogenesis, but also comprehend the molecular basis for novel combined therapy for breast cancer including RSV [158]. 

#### 4.3.2. Epigallocatechin 3-Gallate

A very recent study [159] investigated whether green tea-derived EGCG could modify adipose-derived mesenchymal stem cell differentiation into adipocytes and eventually alter the secretome profile and paracrine regulation of the triple negative breast cancer invasive phenotype. EGCG was able to antagonize the increase of lipoprotein lipase, adiponectin, leptin, fatty acid synthase and fatty acid binding protein. This study suggests that dietary catechin-mediated interventions could inhibit adipogenesis and modulate the adipocytes secretome profile, thus preventing an inflammatory microenvironment able to favor TNBC progression.

Stearic acid, a prototypic saturated fatty acid, stimulated Akt-dependent activation of NF-κB resulting in increased levels of pro-inflammatory mediators [TNF-α, IL-1β, COX-2] in macrophages leading, in turn, to the induction of aromatase in breast cancer models [160]. Several polyphenols including EGCG blocked these inductive effects of stearic acid. Zyflamend, a widely used polyherbal preparation that contains numerous polyphenols including EGCG, suppressed levels of phospho-Akt, NF-κB binding activity, pro-inflammatory mediators and aromatase in the mammary gland in a mouse model of obesity. 

EGCG suppressed tumor growth in a murine breast cancer model, which was associated with decreased Tumor-Associated Macrophages (TAMs) and M2 protumorigenic macrophage infiltration. Expression of chemokines for monocytes was low in tumor cells from EGCG-treated mice, decreased IL-6 and Transforming Growth Factor-β (TGF-β) and increased TNF-α. 

The NF-κB pathway is an important regulator of inflammation and immunity; NF-κB activation is controlled by IKK [IkappaB (inhibitor of NF-kappaB) kinase] complex, which regulates NF-κB activation in response to pro-inflammatory stimuli. Ex vivo incubation of isolated tumor cells with EGCG inhibited the colony-stimulating factor 1 (CSF1), which is involved in breast cancer progression through inducing monocyte differentiation and homing. Moreover, ex vivo incubation of TAMs with exosomes from EGCG-treated 4T1 cells led to the IKKα suppression; the increase of IL-6 and TGF-β; and the decrease of TNF-α. EGCG up-regulated miR-16 in 4T1 cells and in the exosomes. Treatment of breast tumor cells or TAMs with exosomes, derived from EGCG-treated and miR-16-knock-downed 4T1 cells, restored the above effects on chemokines, cytokines, and NF-κB pathway elicited by EGCG-treated exosomes. Authors suggest a novel mechanism by which EGCG exerts anti-tumor activity via regulation of TAMs in TME [161]. 

#### 4.3.3. Retinoids

Retinoids, binding both receptors RARs and RXRs, exert anti-inflammatory activity. Papi et al. tested the new synthetic rexinoid 6-OH-11-O-hydroxyphenanthrene (IIF), in combination with ATRA, reduced survival in mammospheres generated from human tumor specimens and from MCF-7 cells, but not in mammospheres obtained from normal mammary tissues. These effects derived from the inhibition of the inflammatory NF-κB/IL-6 axis which was activated in tumor mammospheres. Moreover, blocking NF-κB axis these retinoids reduced the expression of stemness-related genes, such as Slug, Notch-3, Jagged-1 [162]. Interestingly, the same authors demonstrated that low doses of both RXR and PPARγ ligands synergistically reduced breast cancer stem cell growth dampening the expression of two NF-kB regulated genes and IL6, thus exerting anti-inflammatory activities [163]. Conversely, the addition of ATRA to doxorubicin and entinostat, an inhibitor of histone deacetylase, induces pro-inflammatory genes in triple negative breast cancer cells, potentiating immunostimulatory capacity which leads to tumor regression [164]. 

#### 4.3.4. Omega-3 Polyunsaturated Fatty Acids

Due to their potent anti-inflammatory effects, omega-3 PUFAs, as well as their derivatives, are a promising and safe dietary intervention in reducing breast cancer risk and progression [111,165]. The molecular basis of the inflammatory process in inflammatory breast cancer could be sustained by the regulation of pro-inflammatory cytokines in the crosstalk between cancer cells and tumor stroma. Specifically, in the context of breast TME, TAMs are a significant component of the inflammatory infiltrate in breast tumors, producing different cytokines in relation to their polarization state, and extensive infiltration of TAMs has been linked to poor prognosis in breast cancer [166,167]. Recently, it has been reported that two DHA metabolites, DHEA and DHA-5-HT, attenuated cytokine secretion by TAMs, generated from macrophages co-cultured with breast cancer cells, emphasizing the potential of such compounds in targeting inflammation in breast cancer. [72]. Interestingly, Chas et al. evaluated the concentrations of PUFAs in breast adipose tissue collected from patients with invasive breast cancer. They reported that low levels of EPA are associated with inflammatory breast cancer supporting the link between dietary behaviors and the clinical feature of breast cancer [168]. In cancer cells, the modulation by omega-3 PUFAs of molecules, signals and networks involved in the inflammation [140], underlines the beneficial role of such compounds on counteracting this process that confers breast tumor survival.

### 4.4. Angiogenesis

Most of the tumorigenic pathways are dependent on the deregulation of processes, such as angiogenesis, consisting in the growth of new blood vessels from an existing vasculature, that facilitates tumor growth and progression [169]. Thus, angiogenesis is considered a crucial hallmark of cancer, since it enables pathological tumor metabolism, microenvironmental disruption and tumor cell invasion/metastasis. A wide variety of signaling molecules secreted by cancer cells and cell components within TME have been identified as important players in tumor angiogenesis, among which the vascular endothelial growth factor (VEGF), extracellular proteases, chemokines and cytokines are important targets for anti-angiogenic therapy in breast cancer. Antiangiogenic strategies mainly aim to inhibit early stages of vessel construction and/or to normalize newly formed vessels. 

#### 4.4.1. Resveratrol

Interesting studies indicate lower tumor growth and decreased angiogenesis in MDA-MB-231 tumors in RSV-treated nude mice compared with controls. In vitro experiments show in RSV-treated MDA-MB-231 cells significantly reduced extracellular levels of VEGF, a signaling protein produced by many cell types that stimulates the formation of blood vessels [116]. 

Modulating the metabolic pathway is a novel strategy for controlling tumor angiogenesis and tumor growth. RSV exhibits anti-angiogenic property by targeting the miRNAs such as miRNA-21, miRNA-221/222 and miRNA-27, which are prognostic markers in TNBCs. Using RSV to target miRNAs, which in turn suppresses tumor angiogenesis, should have the potential to inhibit tumor growth, progression, invasion and metastasis and may be developed into an effective therapeutic strategy for the treatment of many different cancers where tumor angiogenesis plays a significant role in tumor growth and progression [170].

Recently it has been reported that RSV downregulates the expression of VEGF, hypoxia-induced factor (HIF-1) and inhibits tumor growth of human breast cancer cells in a nude mouse xenograft model [171]. Relevant results were also published regarding the combined therapy of RSV with thymoquinone which induced geographic necrosis, enhanced apoptosis and decreased VEGF expression. Serum levels of Interferon-gamma (IFNγ) were elevated in mice treated with combination therapy with no liver or kidney toxicity. The anticancer effect of this combination is mediated by apoptosis induction, angiogenesis inhibition and immune modulation [78].

Tristetraprolin (TTP), an mRNA-binding protein, is one of the key proteins that participate in regulating cytokine expression. RSV promoted TTP expression at both the mRNA and protein level in a dose- and time-dependent manner. In addition, the expression of COX-2 and VEGF were significantly suppressed by RSV, while that of inducible nitric oxide synthase (iNOS) was upregulated. Lastly, the effects of RSV on both MCF-7 proliferation and expression of COX-2, VEGF and iNOS were significantly inhibited by TTP knockdown, indicating that TTP mediates the anticancer properties of RSV. In conclusion, RSV can inhibit the proliferation of MCF-7 cells by TTP upregulation, which is associated with downregulation of important mediator of angiogenesis [172].

#### 4.4.2. Epigallocatechin 3-Gallate 

Tumor angiogenesis driven by metabolic reprogramming of endothelial cells is crucial for tumor progression and metastasis in many different cancers, including breast cancers, and has been linked to aberrant miRNA expression profiles. Activation of EGFR signaling pathways is associated with angiogenesis. Different breast cancer cell models display autocrine activation of TGF-alpha/EGFR signaling and produce high levels of VEGF. Treatment with EGCG inhibited the constitutive activation of the EGFR, Stat3 and Akt pathways. These changes were associated with inhibition of VEGF promoter activity and cellular production of VEGF. An analysis of alternative pathways indicated that EGCG strongly inhibited the constitutive activation of NF-κB in breast cancer cell models, and an NF-κB inhibitor strongly inhibited VEGF production. These results suggest that EGCG inhibits VEGF production by inhibiting both the constitutive activation of Stat3 and NF-kappa B, but not ERK or Akt, in these cells. Thus, EGCG may be useful in treating breast carcinoma because it can exert both antiproliferative and antiangiogenic activities [173]. Research studies have investigated the in vivo inhibitory effect of a nutrient mixture containing lysine, proline, arginine, ascorbic acid, but also ECGC on the growth of human breast cancer xenografts in female athymic nude mice. Five to six week old female mice were inoculated with breast cancer cells MDA-MB-231. After injection, mice were fed a regular diet or a regular diet supplemented with 0.5% of the nutrient mixture. The results of this study demonstrated that the nutrient mixture containing EGCG significantly suppressed tumor growth of breast cancer cells in female athymic nude mice and significantly inhibited Matrix MetalloProteinase (MMP) expression, angiogenesis and invasion in breast cancer cells, in vitro, offering promise for therapeutic use in the treatment of breast cancer [174]. 

#### 4.4.3. Retinoids 

Controversial data are reported on the role of retinoids in the angiogenesis of cancer cells. Although in vivo assay showed that ATRA and 9-cis retinoic acid were effective inhibitors of angiogenesis [175], treatment of breast cancer cells with 9-cis retinoic acid induced the expression of many endothelial genes, including vascular endothelial (VE) cadherin. Specifically, the protein SRY-Box Transcription Factor 9 (SOX9) and the ets-family member ER81 are required for the upregulation of VE-cadherin induced by retinoids. The same authors also demonstrated the molecular mechanism by which 9-cis retinoic acid exerts endothelial transdifferentiating properties in breast cancer cells, indicating the involvement of TGFβ signaling pathway in this process [176]. While decreasing cell growth, triggering apoptosis and exerting anti-inflammatory effects, retinoids are able to induce deleterious differentiation endothelial pathway of angiogenesis which potentially represents a negative prognostic indicator of patient outcomes in breast cancers.

#### 4.4.4. Omega-3 Polyunsaturated Fatty Acids

Omega-3 PUFAs may exert their anticancer actions by influencing multiple targets implicated in various stages of the onset and progression of breast cancer, including cell proliferation, cell survival, inflammation and angiogenesis. EPA and DHA have potent anti-angiogenic effects, inhibiting the production of many mediators of the angiogenic cascade, such as VEGF, Platelet-Derived Growth Factor (PDGF), Platelet-Derived Endothelial Cell Growth Factor (PDECGF), COX-2, PGE2, nitric oxide (NO), NFkB, MMP and beta-catenin [177]. Interestingly, DHA represses angiogenesis through the modulation of several proangiogenic expression genes and miRNA contents from breast cancer cells and their exosomes [178]. More recently, the promoting effects on angiogenesis of endothelial cells induced by breast tumor cell-derived exosomes were reversed by DHA treatment highlighting the anti-angiogenetic properties of DHA [179]. As previously reported, omega-3 PUFAs have been shown to improve efficacy also against the angiogenesis of conventional chemotherapies used for breast cancer treatment. Specifically, EPA and DHA potentiate docetaxel activity in remodeling tumor vasculature of mammary tumors [180], opening a window of opportunity in the clinical strategy for breast cancer patients.

## 5. Conclusions

**Figure 5:**
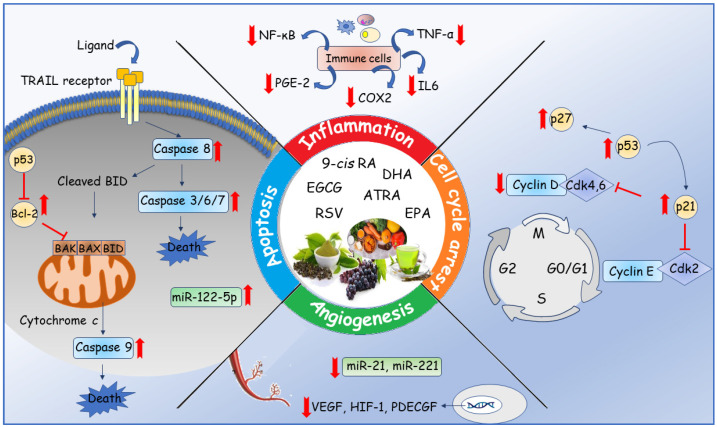
A schematic illustration showing the potential anti-tumoral effects exerted by the nutraceuticals of the MD in the breast cancer progression. Bcl-2: B-cell lymphoma 2; CDK: cyclin-dependent kinase; COX-2: cyclooxygenase 2; HIF-1: hypoxia-inducible factor; IL 6: interleukin 6; miR: microRNA; NF-κB: nuclear factor kappa-light-chain-enhancer of activated B cells; TNF-α: tumor necrosis factor alpha; PDECGF: platelet-derived endothelial cell growth factor; PGE-2: prostaglandin E2; TRAIL: TNF-related apoptosis-inducing ligand; VEGF: vascular endothelial growth factor.

In conclusion, many dietary compounds contained in MD exhibit pleiotropic, multi-target activities and are emerging as promising molecules against breast cancer. Several studies have proposed the potential capability of some nutraceuticals to target important hallmarks of cancer cells, such as cell cycle, apoptosis, inflammation and angiogenesis, underlining the complexity of the molecular mechanisms involved in the anticancer properties by these compounds. Taken together, the data presented in this review show the ability of RSV, EGCG, retinoids and omega-3 PUFAs to affect breast cancer growth and progression (Figure 5), suggesting these nutraceuticals as innovative anticancer candidates. Although the use of these molecules as a therapeutic intervention in a clinical setting is backed by strong biological hypotheses, a clear need appears for developing robust well-designed clinical studies, evaluating the benefits of nutraceuticals from MD. Testing these compounds in combination with chemo- and radio-therapeutic regimens could enhance the efficacy as well as the tolerability of conventional anticancer therapies, improving the clinical outcome and survival of breast cancer patients.

## Data Availability

Not applicable.
